# Transmission Dynamics of Shiga Toxin-Producing Escherichia coli in New Zealand Cattle from Farm to Slaughter

**DOI:** 10.1128/AEM.02907-20

**Published:** 2021-05-11

**Authors:** A. Springer Browne, Anne C. Midwinter, Helen Withers, Adrian L. Cookson, Patrick J. Biggs, Jonathan C. Marshall, Jackie Benschop, Steve Hathaway, Lynn Rogers, Shahista Nisa, Carter R. Hranac, Taylor Winkleman, Nigel P. French

**Affiliations:** a ^*m*^EpiLab, School of Veterinary Science, Massey University, Palmerston North, New Zealand; b Ministry for Primary Industries, Wellington, New Zealand; c AgResearch Limited, Palmerston North, New Zealand; d New Zealand Food Safety Science & Research Centre, Palmerston North, New Zealand; e School of Fundamental Sciences, Massey University, Palmerston North, New Zealand; INRS—Institut Armand-Frappier

**Keywords:** Shiga toxin-producing *Escherichia coli*, genomics, MALDI-TOF, One Health, cattle

## Abstract

Cattle are asymptomatic carriers of Shiga toxin-producing Escherichia coli (STEC) strains that can cause serious illness or death in humans. In New Zealand, contact with cattle feces and living near cattle populations are known risk factors for human STEC infection. Contamination of fresh meat with STEC strains also leads to the potential for rejection of consignments by importing countries. We used a combination of PCR/matrix-assisted laser desorption ionization–time of flight mass spectrometry (MALDI-TOF) and whole-genome sequencing (WGS) to evaluate the presence and transmission of STEC on farms and in processing plants to better understand the potential pathways for human exposure and thus mitigate risk. Animal and environmental samples (*n* = 2,580) were collected from six farms and three meat processing plants in New Zealand during multiple sampling sessions in spring of 2015 and 2016. PCR/MALDI-TOF analysis revealed that 6.2% were positive for “Top 7” STEC. Top 7 STEC strains were identified in all sample sources (*n* = 17) tested. A marked increase in Top 7 STEC prevalence was observed between calf hides on farm (6.3% prevalence) and calf hides at processing plants (25.1% prevalence). Whole-genome sequencing was performed on Top 7 STEC bacterial isolates (*n* = 40). Analysis of STEC O26 (*n* = 25 isolates) revealed relatively low genetic diversity on individual farms, consistent with the presence of a resident strain disseminated within the farm environment. Public health efforts should focus on minimizing human contact with fecal material on farms and during handling, transport, and slaughter of calves. Meat processing plants should focus on minimizing cross-contamination between the hides of calves in a cohort during transport, lairage, and slaughter.

**IMPORTANCE** Cattle are asymptomatic carriers of Shiga toxin-producing E. coli (STEC) strains, which can cause serious illness or death in humans. Contact with cattle feces and living near cattle are known risk factors for human STEC infection. This study evaluated STEC carriage in young calves and the farm environment with an in-depth evaluation of six farms and three meat processing plants over 2 years. An advanced molecular detection method and whole-genome sequencing were used to provide a detailed evaluation of the transmission of STEC both within and between farms. The study revealed widespread STEC contamination within the farm environment, but no evidence of recent spread between farms. Contamination of young dairy calf hides increased following transport and holding at meat processing plants. The elimination of STEC in farm environments may be very difficult given the multiple transmission routes; interventions should be targeted at decreasing fecal contamination of calf hides during transport, lairage, and processing.

## Abstract (precis)

Cattle are asymptomatic carriers of Shiga toxin-producing E. coli (STEC) strains, which can cause serious illness or death in humans. Contact with cattle feces and living near cattle are known risk factors for human STEC infection.

## INTRODUCTION

Shiga toxin-producing Escherichia coli (STEC) strains are globally estimated to cause 2.8 million cases of illness, 3,890 cases of hemolytic uremic syndrome (HUS), and 230 deaths annually (1). Illness and acute kidney failure are more common in young children (2). New Zealand has a relatively high incidence of notified STEC infection in humans, with 11.4 STEC cases per 100,000 population reported in 2017 (3). There has been a general increase in the incidence of STEC cases since 1997 (3).

Cattle are the primary reservoir of STEC, in which the bacteria colonize the intestine and are excreted in fecal material (4). Due to detection of STEC in raw ground beef, and following outbreaks associated with consumption of undercooked beef patties, the United States declared STEC O157 an adulterant of beef in 1994, followed by the declaration of 6 additional serogroups (O26, O45, O103, O111, O121, and O145) as adulterants in late 2011 (5). Mandatory testing for the additional six STEC serogroups began on 5 March 2012 (5). These seven serogroups are commonly known as the “Top 7” STEC. In 2016 and 2017, 50% of New Zealand beef exports, valued at $1.16 billion NZ, were sent to the United States (6).

Previous research in New Zealand, as well as in other countries, has identified calves as having a higher prevalence of STEC than adult cattle (7–9). A previous randomly stratified cross-sectional study of 102 New Zealand farms from the six largest dairy regions revealed a relatively high prevalence of Top 7 STEC carriage by calves; in total, 20.3% of dairy calves and 75.0% of the farms tested positive for at least one of the Top 7 STEC strains (10). In New Zealand, very young dairy calves that are surplus to replacement needs are slaughtered between the ages of 4 to 10 days, and the meat is sold as veal for human consumption; these calves are called bobby calves.

This study evaluated the risk factors that are likely to contribute to contamination of veal carcasses and the consequential foodborne risks to consumers. Whole-genome sequencing (WGS) was used to investigate the transmission of defined STEC strains on farms to animals and the environment, and to determine the between- and within-farm genetic variation of STEC strains in the farm environment over time.

## RESULTS

### Top 7 STEC prevalence determined using NeoSEEK.

Real-time PCR (RT-PCR) testing determined 37.3% (95% confidence interval [CI], 35.4 to 39.2) of samples (*n* = 2,580) were potential STEC strains (*eae*, *stx_1_*, or *stx_2_* positive). The overall prevalence for any of the Top 7 STEC strains was estimated to be 6.2% (95% CI, 2.1 to 10.3). Prevalence varied by STEC serogroup (Table 1), with STEC O103, STEC O26, and STEC O145 having the highest prevalences, whereas STEC O121 was not detected from the farm and processing plant environments, and STEC O111 was very rare.

**TABLE 1:** Prevalence of Top 7 STEC by NeoSEEK assay for all animal and environmental samples evaluated (*n* = 2,580)

Serogroup	STEC prevalence (% [95% CI])
O103	2.7 (0–5.5)
O111	0.2 (0–0.01)
O121	0
O145	1.7 (0–4.0)
O157	0.5 (0–1.7)
O26	2.2 (0–4.6)
O45	0.6 (0–1.9)

The Top 7 STEC prevalence estimates for each farm in the study are provided in Table 2, with prevalence data for each Top 7 STEC serogroup by farm presented in Table S3 in the supplemental material. Prevalence data from both years and all five periods are shown in Table 3, calculated using a general linearized model with farm and sample source as random effects. The prevalence of Top 7 STEC within each farm varied significantly between farms (*P* < 0.0001). Overall Top 7 STEC detection significantly increased between 2015 and 2016 (Table 3) [χ^2^ (1) = 5.4; *P* = 0.02], but this may have been influenced by the change in DNA processing from the Kingfisher flex purification system in 2015 to a double-wash boil preparation in 2016. STEC prevalence was not significantly related to the five periods of the study (Table 3) (Fisher’s exact test, *P* = 0.87), or the three active calving periods (early, middle, and late) of the calving season [χ^2^ (2) =1.12; *P* = 0.57].

**TABLE 2:** Prevalence of Top 7 STEC in all animal and environmental sources in each farm^*a*^

Farm identifier (*n*)	Prevalence (% [95% CI])
F1 (368)	10.2 (6.5–15.8)
F2 (483)	2.8 (1.5–5.0)
F3 (433)	7.0 (4.4–11.1)
F4 (317)	2.0 (0.9–4.5)
F5 (491)	5.8 (3.6–9.4)
F6 (488)	6.8 (4.3–10.8)

a Estimated using generalized linear models with calving period and sample source included as random effects.

**TABLE 3:** Prevalence of Top 7 STEC in all animal and environmental sources on all study farms over time (year and period)^*a*^

Year or period (*n*)	Prevalence (% [95% CI])
Year	
2015 (1,244)	3.8 (2.4–5.8)
2016 (1,336)	7.6 (5.2–10.9)
Period	
Precalving (60)	2.6 (0.7–8.8)
Early calving (909)	4.2 (2.7–6.5)
Middle calving (829)	5.0 (3.2–7.8)
Late calving (722)	5.3 (3.5–8.3)
Postcalving (60)	4.6 (1.6–12.0)

a Estimated using generalized linear models with farm and source of sample included as random effects.

Top 7 STEC strains were identified by the NeoSEEK method in all sample sources (*n* = 17) tested in this study (Table 4). Both calf and cow colonization samples had a significantly lower prevalence of Top 7 STEC [χ^2^ (17) = 88.5; *P* < 0.0001] than that in samples from calf pens and calf hides. On farm, younger calves (aged 1 to 3 days) had a lower prevalence in recto-anal mucosal swabs (RAMS) (0.4%) and on calf hide on farm (1.7%) than older calves (aged 4 to 10 days; RAMS, prevalence 3.5%; calf hide on farm prevalence, 6.6%).

**TABLE 4:** Top 7 STEC prevalence of all sample sources (*n* = 17)^*a*^

Sample type	Sample source	Prevalence (% [95% CI])
Animal (*n* = 6)	RAMS: calf on farm (*n* = 553)	1.6 (0.8–3.2)
	Hide: calf on farm (*n* = 553)	3.4 (1.9–5.8)
	Hide: calf at processing plant (*n* = 186)	23.8 (15.7–34.5)
	Preintervention calf carcass (*n* = 186)	7.7 (4.2–13.6)
	RAMS: cow on farm (*n* = 290)	0.5 (0.1–2.1)
	Dam udder sponge swab (*n* = 290)	1.0 (0.4–2.9)
Environmental (*n* = 11)	Calf pen overboot (pre- or post-calving period) (*n* = 24)	3.6 (0.5–21.9)
	Calf pen overboot (early, middle, and late calving period) (*n* = 92)	16.7 (9.4–28.1)
	Feedpad overboot (*n* = 7)	11.1 (1.4–52.3)
	Paddock overboot (*n* = 59)	8.4 (3.5–18.7)
	Pen colostrum sponge sample (*n* = 91)	2.7 (0.8–8.5)
	Pen concentrates sponge sample (*n* = 10)	21.9 (6.2–54.5)
	Pen water trough sponge sample (*n* = 23)	10.8 (3.2–31.0)
	Water and concentrate sponge sample (pre- or post-calving period) (*n* = 24)	3.6 (0.5–21.9)
	Milk filter (*n* = 36)	8.9 (3.1–23.1)
	Bird feces (*n* = 60)	5.4 (1.9–14.3)
	Effluent (*n* = 60)	12.8 (6.2–24.6)

a Estimated using generalized linear models with farm and calving period included as random effects.

Of 186 bobby calves sampled from farm to processing plant, there was an increase of hide prevalence between the farm and processing plant from 6.3% to 25.1% (Table 5).

**TABLE 5:** Top 7 STEC prevalence of calves (*n* = 186) sampled from farm to processing plant^*a*^

Sample source	Prevalence (% [95% CI])
RAMS: calf on farm	3.0 (1.2–7.3)
Hide: calf on farm	6.3 (3.1–12.5)
Hide: calf at processing plant	25.1 (15.3–38.3)
Preintervention calf carcass	8.2 (4.1–15.6)

a Estimated using generalized linear models with farm and calving period included as random effects.

### Factors associated with STEC hide contamination, carcass contamination, and fecal carriage of calves.

**(i) Independent evaluation of outcome variables for calf colonization, hide contamination, and preintervention carcass contamination.** The presence of at least one positive calf RAMS sample in a pen was associated with hide contamination in animals in the same pen, and the presence of at least one positive calf hide in a shed was strongly associated with an increased risk of a calf in that shed being detected as colonized on the day of sampling. However, in the shed, a calf whose hide was contaminated was not necessarily detected as colonized (see Table S1 in the supplemental material), while at the processing plant, if a calf’s hide was contaminated with Top 7 STEC, this was not significantly associated with contamination of its carcass.

Factors associated with Top 7 STEC colonization and hide contamination of calves on dairy farms are shown in Table 6. Calves were more likely to be colonized when the proportion of calves within the same shed with contaminated hides increased (odds ratio [OR] = 2.59, per 10% increase in prevalence). The proportion of Top 7 STEC-positive colonized calves in the shed was associated with positive hide prevalence (OR = 3.36, per 10% increase in shed prevalence).

**TABLE 6:** Generalized linear models of risk factors for Top 7 STEC on farm and in processing plants

Model or outcome variable	Top 7 STEC random effect variance	Risk factor	OR	95% CI	Pr(>|*z*|)
Model A					
Top 7 STEC calf colonization (RAMS) on farm	Farm (0.29)	Proportion of Top 7 STEC-positive calf hide in same shed (per 10% increase in prevalence)	2.59	2.0, 3.2	0.002^*a*^
Model B					
Top 7 STEC on calf hide on farm	Farm (0.38)	Proportion of Top 7 STEC-positive RAMS from calves in same shed (per 10% increase in prevalence)	3.36	2.1, 5.5	<0.0001^*a*^
Model C					
Top 7 STEC on calf hide at processing plant	Farm (0.21)	Top 7 STEC-positive RAMS from cow on the same farm visit	14.7	3.3, 64.8	0.0004^*a*^
		Plant hide fecal score^*b*^	0.04^*a*^
		Plant hide fecal score is 2 compared to 1	3.32	1.29, 8.6	0.01^*a*^
		Plant hide fecal score is 3 compared to 1	4.76	1.4, 16.6	0.01^*a*^
		Plant hide fecal score is 4 compared to 1	4.69	0.9, 24.2	0.06
Model D					
Top 7 STEC on preintervention calf carcass at processing plant	Farm (0.0)	No. of farms visited by calf truck	1.1	1.00, 1.20	0.01^*a*^
		Top 7 STEC-positive calf hide sample on farm on the same farm visit	4.53	1.02, 1.21	0.02^*a*^
		No. of calves in calf truck	0.98	0.97, 1.00	0.02^*a*^

a Significant variable (*P* < 0.05).

b Likelihood ratio test *P* value of variable as a whole.

**(ii) Factors associated with contamination of calf hides at processing plants and of preintervention calf carcasses.** Contamination of calf hides with Top 7 STEC at the processing plant was associated with at least one positive RAMS cow sample on farm (OR = 14.7) and increased visual contamination of the hide (fecal score of 2 compared to fecal score of 1, OR = 3.32; fecal score of 3 compared to fecal score of 1, OR = 4.76) (Table 6).

Evaluation of preintervention calf carcasses at the processing plant indicated that “increased number of farms visited by the calf transport truck” was associated with positive Top 7 STEC results (OR = 1.1, per increase in one farm visited by transport truck) (Table 6). Having adjusted for confounding by the number of farms visited, the number of calves in each truck was negatively associated with contamination of preintervention carcasses (OR = 0.98 per calf).

### Bacterial isolation.

The overall success of bacterial isolation of a detected Top 7 STEC serogroup from the samples positive via the NeoSEEK assay was 14.2% (29/204) (see Table S2 in the supplemental material).

### Whole-genome sequencing analyses of bacterial isolates.

Twenty-five isolates belonging to serogroup O26, which was the most prevalent and widely distributed serogroup retrieved from all farms (*n* = 6) and several sources (*n* = 8) (see Data Set S1 in the supplemental material) underwent whole-genome sequencing. Serogroup O26 genetic analysis was conducted using three measures of genetic variation, as follows: the core genome (based on single nucleotide polymorphisms between core genes); the accessory genome (presence or absence of genes not present in all isolates); and virulence factor genes. Two potential sources of variation were considered, farm and isolation source (Table 7). “Farm” was the strongest determinant of genetic variation; isolates recovered from the same farm were much more similar than isolates compared between farms. “Farm” explained 75% to 87% of the genetic variation, whereas the sample type/isolation source explained very little of the variation once “farm” had been taken into consideration.

**TABLE 7:** PERMANOVA analysis of core genome, accessory genome, and virulence factors of O26 isolates (*n* = 25) by farm and isolation source

Factor evaluated (*n*)	df	Pseudo-*F*	*P*	Component of variation (%)^*a*^
Core genome (*n* = 1,974 SNPs)
Farm (6)	4	17.17	0.0001	83.9
Isolation source (8)	6	1.05	0.42
Accessory genome (*n* = 2,265 genes)
Farm (6)	4	10.64	0.0001	75.6
Isolation source (8)	6	0.996	0.47
Virulence genes (*n* = 28 genes)
Farm (6)	4	19.53	0.0004	86.7
Isolation source (8)	6	0.97	0.40

a Residual variation was as follows: core (16.1%), accessory (24.4%), and virulence (13.3%).

**FIG 1:**
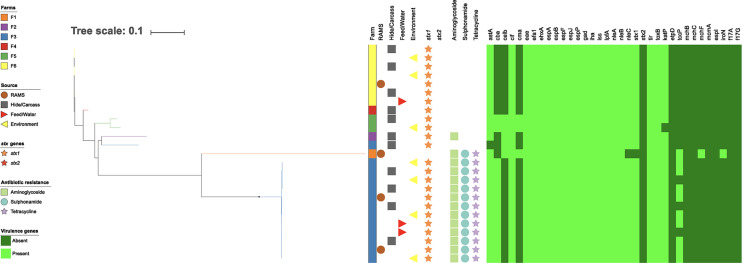
Core genome single-nucleotide polymorphism (SNP) RAxML phylogenetic tree of E. coli serogroup O26 genomes (*n* = 25; n = 1,974 SNPs detected) annotated by farm, source, antibiotic resistance gene class, and virulence genes.

**FIG 2:**
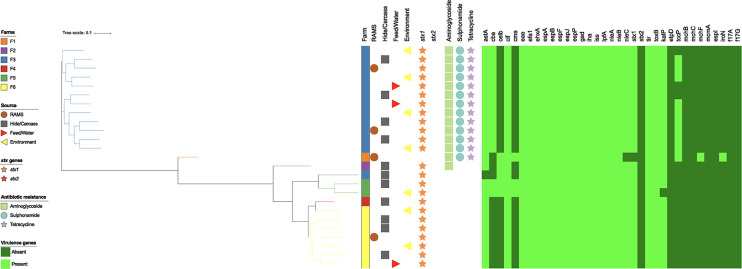
RAxML phylogenetic tree of E. coli serogroup O26 genomes (*n* = 25) constructed using the presence or absence of accessory genome content (*n* = 2,265 accessory genes detected), annotated by farm, source, antibiotic resistance gene class, and virulence genes.

**FIG 3:**
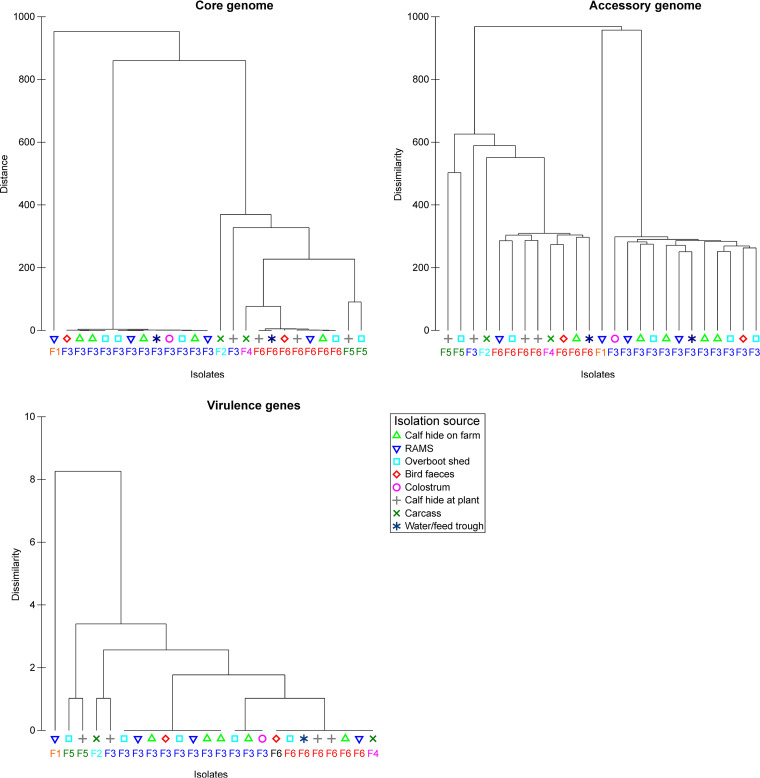
Hierarchical cluster analysis of serogroup O26 genomes (*n* = 25) by source (*n* = 6) and farm (*n* = 8; labeled F1 through F6) for core genome (1,974 SNPs detected), accessory genome (*n* = 2,265 genes detected), and virulence genes (*n* = 28 genes detected).

The population structure was visualized using dendrograms. Phylogenetic trees of the core and accessory genomes for serogroup O26 (Fig. 1 and 2), and non-O26 serogroups (see Fig. S1 in the supplemental material) are annotated with antibiotic resistance gene classes and virulence factor genes identified in isolates. Hierarchical cluster analysis was performed for the serogroup O26 isolates with farm and isolation source (Fig. 3) as factors. Figures 1 and 2 show clear differentiation of serogroup O26 E. coli strain based on farm but not isolation source, consistent with the permutational multivariate analysis of variance (PERMANOVA) analysis summarized in Table 7.

## DISCUSSION

The aim of this study was to explore the presence and transmission of STEC on farms and in processing plants to better understand the pathways that lead to human exposure and thus potential risk mitigation measures.

### Prevalence and transmission routes of STEC in environmental and animal samples on farm.

Every source, both animal and environmental (*n* = 17), was positive for Top 7 STEC by the NeoSEEK STEC confirmation assay. The estimated prevalence of Top 7 STEC-colonized calves and cows was relatively low (0.5 to 1.6%) compared to environmental factors such as the calf pen floor (16.7%). However, although we used a modeling technique that incorporated random effect terms (e.g., farm, calving period, and source) in order to correct for the clustering of animals and environments within farms, the results should be interpreted with caution due to the concurrent analysis of multiple sources and variation in sampling of these sources between farms and time periods.

Evaluation of factors associated with Top 7 STEC calf colonization and calf hide contamination on farm, combined with the genomic analysis, indicated that transmission between calves, between the environment and calves, and between cows and calves, were all highly likely. However, the direction of transmission could not be determined without more intensive longitudinal sampling.

In this study, 5.4% of bird fecal samples tested positive for Top 7 STEC strains, and two STEC O26 isolates were obtained. European starlings (Sternus vulgaris) can shed STEC O157, with transmission to calves within 3 days during experimental conditions (11). Bird droppings sampled from two farms 32.5 km apart found the same restriction endonuclease digestion (REDP) subtype of O157:H7, indicating birds as a possible vector (12). In our study, since bird fecal samples were taken from surfaces in calf pens and not directly from birds, these results should be interpreted with caution, as dust present in calf pens has also been shown to be STEC positive (13).

Hide contamination in the calf pen was strongly associated with the proportion of colonized calves positive in that pen on the same day of sampling (OR = 3.36, per 10% increase in RAMS-positive prevalence). This finding, while intuitively obvious, indicates that active shedding of Top 7 STEC by calves in calf pens is closely linked to hide contamination. Increased shedding of E. coli O157 was also associated with hide contamination of calves (14), while detection of a non-O157 STEC serogroup in a fecal sample was associated with positive prevalence in a hide sample in culled dairy cattle (15).

Whole-genome sequencing (WGS) analysis of 40 bacterial isolates revealed that isolates from multiple sources on the same farm displayed a high degree of genetic similarity, consistent with widespread dissemination of clonal strains across multiple environments within the same farm (Fig. 1 to 3). One clear example of clonal transmission was seen at farm 3 (F3), where 12 STEC O26 isolates from eight animal and environmental sources (Fig. 1) were isolated in 2015 over three periods of the calving season. This provides evidence of persistence and widespread dissemination of dominant strains of STEC in the dairy farm environment. In a study in the United States, similar pulsed-field gel electrophoresis (PFGE) profiles were seen year to year in the same feedlot, with little to no evidence of transmission of organisms between pens in each feedlot, indicating highly localized transmission dynamics (16). In New Zealand, PFGE analysis of E. coli serogroup O26 bacteria found that isolates from the same farm clustered more closely than isolates from different farms (17). The marked difference between isolates on different farms may also indicate strong biosecurity measures on New Zealand farms that prevent interfarm transmission of STEC.

### Prevalence and transmission routes of STEC on hide and preintervention veal carcasses at processing plants.

A large increase in prevalence was noted between Top 7 STEC-positive calf hides on farm (6.3%) and those at the processing plant (25.1%), indicating that extensive cross-contamination occurred during calf transport and lairage. This finding has been noted in another study where STEC O157 hide prevalence increased from 50.3% to 94.4% between the times cattle were loaded for transport and stunned at the processing plant (18).

Postslaughter, 8.2% of preintervention calf carcass samples were positive for Top 7 STEC by the NeoSEEK assay. Other research has found that the prevalence of STEC contamination of calf hides and carcasses may differ dependent on the processing plant and interventions adopted. A U.S. study of young veal calves at processing plants isolated STEC O157 bacteria from 20% of hides and 7% of preintervention carcasses (19).

Top 7 STEC on calf hides at the processing plant was associated with colostrum cow STEC colonization (RAMS) on the same day of sampling (OR = 14.7) and increased visual contamination of calf hides at the processing plant (fecal score of 2 compared to 1, OR = 3.32; Table 6). An Australian study found that greater concentrations of STEC O157 on hides were correlated with greater concentrations of STEC O157 on pre-evisceration carcasses (20). The presence of an actively shedding cow may be indicative of widespread dissemination of STEC on the dairy farm.

Top 7 STEC contamination of preintervention carcass samples was associated with an increasing number of farms visited by the transport truck (OR = 1.1, per increase of one farm; Table 6). A range of 10 to 71 farms were visited by a single transport truck during the study period. These findings provide evidence of the importance of hide contamination on farm and cross-contamination of calves during transport to eventual contamination of preintervention carcasses. For every farm visited by the calf transport truck, the chances of an individual calf being contaminated increase; a single calf carrying STEC may have the potential to cross-contaminate many other calves during transport and lairage (21). The increasing number of calves in the truck (range of 52 to 423 calves per truck) was found to be protective in our analysis (OR = 0.98); although difficult to explain biologically, this supports our other genomic findings that the farm is more important than individual animals with regard to STEC contamination. A positive association was found between cattle carcasses that were positive for E. coli O157:H7 and transportation in a truckload that contained at least one high-shedding (greater than 10^4^ CFU/g of feces) cow (22). Analysis in our study indicated that the presence of a Top 7 STEC-contaminated hide on the farm was found to be significantly associated with contamination of preintervention carcass samples (OR = 4.53).

The presence of isolates on calves at the processing plant that were genetically distinct from isolates on their farm of origin is consistent with cross-contamination on hides of calves during transport and lairage. One STEC O26 from a calf hide at the processing plant from farm 3 (F3), as well as an STEC O157 from a preintervention carcass from farm 1 (F1), both showed clear genetic differences from the predominant strains on their respective farms (Fig. 1 to 3). This supports previous New Zealand research that STEC from a calf on a separate farm can directly or indirectly contaminate the hide and carcass of another calf during transport and lairage (21). Other research (using PFGE) found that only 29% of STEC O157 isolates collected from cattle at a processing plant matched isolates collected on the originating farm, indicating cross-contamination of carcasses from other sources (18). This finding provides further evidence of the risk of transport and lairage to increased hide contamination, as well as the risk of contamination with increasing numbers of farms visited by calf transport trucks.

### Representativeness of the study.

Due to the intensive sampling from multiple sources (up to 96 samples a day collected and processed), only a limited number of farms were included in the study (*n* = 6). However, the focused assessment on several farms allowed for an in-depth evaluation of transmission and contamination pathways from farms to processing plants.

Selection bias existed in the Top 7 STEC results from NeoSEEK, since, with the exception of the cohort of calves positive in-plant, only samples that had previously been found potentially positive for Top 7 STEC using RT-PCR screening for virulence genes were eligible for NeoSEEK testing. This sampling strategy was adopted due to resource limitations that meant that all samples (*n* = 2,580) could not be submitted for NeoSEEK testing. The NeoSEEK method is reported to be highly specific for Top 7 STEC, with 86 targets; therefore, many samples that had virulence genes, and were therefore positive on prescreening, were not positive for Top 7 STEC by NeoSEEK. This approach is likely to have resulted in an underestimation of prevalence due to imperfect sensitivities of the prescreening and NeoSEEK assays. However, the impact of prescreening on estimates of prevalence is likely to be small. Evidence for this is provided by the subset of calves that were positive in at least one on-plant sample. In this cohort, all four samples (*n* = 118 calves; 472 samples) were submitted for NeoSEEK analysis, regardless of prescreening. This revealed that 2.4% (5/206) of samples that were negative by prescreening were positive for Top 7 STEC by NeoSEEK (data not shown). This indicates that prescreening is likely to have resulted in a reduction in sensitivity of detection of Top 7 STEC in samples (assuming high specificity of the NeoSEEK assay), but the effect on prevalence estimates is likely to be small. Notably, the Top 7 STEC prevalence results in the present study were similar to a randomized cross-sectional study of all dairy farms in New Zealand that used the NeoSEEK assay for every sample without any prescreening measures (10).

Our molecular detection methods (NeoSEEK and RT-PCR) only allowed for prevalence estimates of STEC, rather than of the concentration of bacteria in the samples. While this may be a limitation, these methods are used to screen and confirm the presence of Top 7 STEC in beef trim for the export market; they are directly relevant to the New Zealand red meat industry.

Finally, our analysis of the effect of transport and lairage of calves to processing plants was limited by not sampling calves from other farms that were transported with our cohort calves. However, our investigation identified increased cross-contamination during transport and lairage, as well as the increasing risk of cross-contamination with each farm visited; this would be an important factor to fully evaluate in further studies.

### Conclusion.

Our study provided evidence that the key factors in colonization of very young calves is a combination of mother-to-calf, calf-to-calf, and environment-to-calf factors. Several mother-related pathways, including cow colonization and contamination of colostrum and milk filters, strongly indicate that cows are part of the transmission cycle. The contamination of calf hides, while indicative of shedding of Top 7 STEC within the pen, may also act as a transmission route, due to calves’ nuzzling behavior with other calves. Our genomic analyses support the conclusion that cows, calves, the environment, and feed and water sources are contaminated or colonized by the same strains of Top 7 STEC, indicating that multiple transmission pathways are in action.

Transport and lairage led to significant increases in both the prevalence and the genomic diversity of Top 7 STEC on calf hides at the plant, indicating that extensive cross-contamination of hides occurs. Visually detectable contamination of hides, as well as contamination of calf hides on farms, increased the level of carcass contamination immediately after hide removal. The increase in the number of farms visited by the transport truck was also associated with an increased level of carcass contamination. This suggests that calf hide contamination or calf colonization with STEC from one farm can lead to significant levels of cross-contamination of calf hides and contamination of the carcasses of calves from other farms.

Due to the large number of potential transmission routes identified in this study, preventing exposure of very young calves to STEC on dairy farms is likely to be very difficult to achieve in practice. Even within the first 3 days of life, calves already had Top 7 STEC hide contamination, and one was already colonized with a Top 7 STEC strain.

Reduced contamination of calf hides may lead to decreased transmission of STEC on farms, as well as a decreased opportunity for initial contamination of carcasses during slaughter and dressing. Decreasing persistence of STEC in the calf pen environment, as well as on transport trucks and in lairage, may further decrease the level of contamination. Sanitizers and local disinfection could be applied, but there would likely be significant practical limitations enlisting farmers to participate. However, several opportunities for chemical interventions exist during transport and lairage, namely, loading into a transport truck, unloading from a transport truck, and while in lairage.

Although application of specific control measures to minimize the level of contamination of hides and fresh carcasses is an important element of risk management, there is still a need for meat hygiene training and the implementation of basic hygiene practices. In 2016, the Meat Industry Association in New Zealand worked together with the Ministry for Primary Industries to introduce nine initiatives aimed at reducing the degree of veal carcass contamination with Top 7 STEC, including hosting workshops targeted at senior operators, supervisors, technical staff, and on-site verification staff (6). Continued educational efforts at meat processing plants in New Zealand are likely to further reduce carcass contamination.

These results indicate that Top 7 STEC strains are likely to be maintained to some degree in the farm environment throughout the year, and there are a number of risk factors that have the potential to increase the level of colonization of young calves on dairy farms, as well as hide contamination and cross-contamination to the carcass in the slaughterhouse. While this research suggests there is limited opportunity to reduce transmission on-farm by controlling individual transmission pathways, it is clear that conditions of transport, lairage, slaughter, and dressing have a profound effect on the level of cross-contamination of the carcass with Top 7 STEC strains, thereby impacting the potential for foodborne transmission.

## MATERIALS AND METHODS

**FIG 4:**
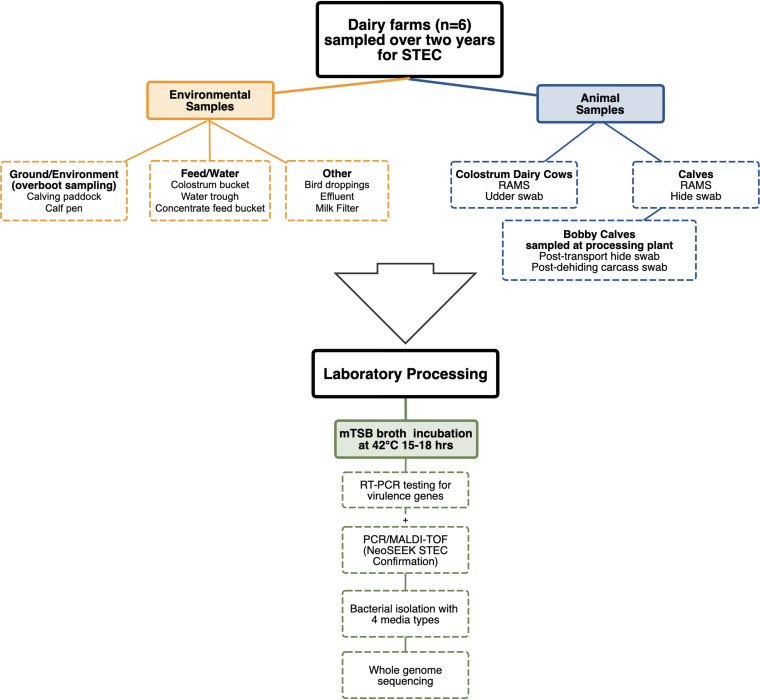
Flow diagram of sampling and laboratory processing methods.

A summary of the sampling and processing methods are illustrated in Fig. 4.

### Farm and meat plant selection.

Six farms in the Waikato region of New Zealand participated in this study during the 2015 and 2016 spring calving seasons (July to September). Selection was determined using previous Top 7 STEC prevalence results from a cross-sectional study carried out in 2014 (10), willingness to participate, and having very young calves processed by two specific meat processing companies. All farms were considered “closed.” meaning they had not received new live animals from any other farm within the past 5 years, and all participated in 100% dairy farming (i.e., no other livestock were present). The size of farms ranged from 230 to 750 milking cows, and the distance between farms ranged from 13 km to 105 km.

We enlisted the participation of two meat processing companies. One company provided access to two veal processing plants for sampling, as well as logistical information regarding calf transport to facilitate planning. The second meat processing company only participated in the study for two sampling periods during 2015, when some of the calves from the selected study farms were sent to this plant for processing.

### Sample collection.

The majority of sampling was performed three times over the calving season, during the periods when the first 0 to 24% of calves were born (early), when 25 to 75% of calves were born (middle), and when the final 76 to 100% of calves were born (late), for each calving year. Sampling targeted very young calves (0 to 3 days of age), bobby calves and replacement calves (4 to 10 days of age), and colostrum cows (postpartum cows that had given birth within the past 4 days). Calves sampled on-farm were also sampled at processing plants. This entailed hide sampling prior to hide removal, and carcass sampling immediately post-hide removal (“preintervention carcass” was defined as before chemical and physical means are used in-plant to decrease bacterial contamination on the carcass). Selected farm environmental samples were collected before and after the calving season and included effluent, bird feces, paddock overboots, calf pen overboots, and drinking water and feeding trough swabs (colostrum and concentrates). For each farm visit, one milk filter was collected after the morning milking concluded and placed in a dry sterile plastic bag.

Samples were collected using Amies swabs (Copan Diagnostics, Inc., Brescia, Italy), sterile cellulose sponge swabs (EZ-Reach sponge sampler in 25 ml buffered peptone water [BPW]; World Bioproducts, Woodinville, WA), and overboots (Envirobootie, premoistened with double-strength skim milk broth; Hardy Diagnostics, CA, USA).

Colonization of calves and cows was determined by using a recto-anal mucosal swab (RAMS) technique with an Amies swab. After determining the age distribution of all calves, up to three calf pens were selected that allowed for the maximum number of calves (*n* = 15) in the two age groups to be sampled, with equal numbers sampled per pen where possible. Sampling was prioritized to ensure that the sample contained a maximum of 10 calves that were being transported to the processing plant that day. Selection was as described in Browne et al. (10). A maximum of 10 cows that had calved within the past 4 days (colostrum herd) were selected for sampling using the same methodology. Any animals that appeared injured or sick, based on visual clinical assessment by the sampler (a registered veterinarian), were excluded from sampling.

Sponge swabs were used for sampling of the calf hide, cow udder, preintervention carcass, and environmental samples (colostrum feeder, water trough, concentrate feed trough, bird feces, and effluent). For calf hide and preintervention carcass sampling, calves were sampled on one median side of the body, from the medial aspect of the knee to the axilla, the entire ventral thorax and abdomen, and the medial aspect of the groin to the hock, using three back-and-forth passes in each area. The median side of the body sampled was rotated between hide on farm, hide at processing plant, and preintervention carcass.

Cow udders were sampled on the ventral aspect of the udder, lateral to each udder and between the teats, using three back-and-forth passes for each region. Colostrum feeders, water troughs, and concentrate feed trough samples were obtained by wiping the entire interior of each container with the sponge swab. Three to five bird fecal droppings were collected from each calf pen sampled on each visit using sterile forceps and placed into a sterile cellulose sponge swab sampling bag. Effluent samples were obtained by inserting the swab into the effluent at a designated location on each farm, chosen for its proximity to daily fecal outflow and safety of obtaining a sample. After sampling, each sponge was secured in its sterile bag and manually massaged to incorporate the 25 ml of BPW into the sample.

Overboots were used to sample calf pens, calving paddocks, and feedpads. Sterile plastic boot covers were placed over the boots before placing the overboots to prevent cross-contamination. The sampler walked the entire perimeter of the sampling area and then zig-zagged in equal transects across the area (six transects for the pens and three transects for the paddock and feedpad). During the transects in the calving paddock and feedpad, the sampler also walked the perimeter of high-traffic areas, such as water troughs or feed areas.

All samples were placed in an ice-filled insulated container in the field and shipped with fresh ice in insulated boxes to *^m^*EpiLab, Massey University (Palmerston North, New Zealand) for processing.

### Sample processing.

All sample types were processed at *^m^*EpiLab the day after collection. Sponge swabs, milk filters, and overboots were stomached with 25 ml modified tryptone soya broth (mTSB; Oxoid, Hampshire, UK); Amies swabs (i.e., RAMS samples) were vortexed with 25 ml mTSB broth. The mTSB samples were incubated at 42°C for 15 to 18 h for enrichment. An aliquot of the enriched broth was stored with glycerol in a 4:1 ratio at −80°C, and another was processed for DNA extraction. DNA processing for the 2015 sampling utilized the Kingfisher flex purification system per the manufacturer’s instructions. A subset of samples collected during 2015 was also processed using a double-wash boil preparation method, according to Neogen’s laboratory instructions. In brief, 1 ml of enrichment broth was centrifuged at 15,000 × *g* for 10 min, supernatant was discarded and the pellet resuspended in 1 ml phosphate-buffered saline (PBS), centrifuged again and the supernatant discarded, and resuspended in 1 ml of water and boiled at 100°C for 10 min. In 2016, all DNA processing was by a double-wash boil preparation method.

Each DNA sample was screened for three virulence genes (*eae*, *stx_1_*, and *stx_2_*) using real-time PCR (RT-PCR) with primers and methods as previously described (23). The limit of detection (LOD) of the virulence assay (RT-PCR screening for all three virulence factors) was estimated to be 9.9 × 10^2^ CFU per ml.

A selection of samples was screened using the NeoSEEK STEC confirmation assay (a PCR/matrix-assisted laser desorption ionization–time of flight mass spectrometry [MALDI] assay), an AOAC-approved (AOAC no. 081901) confirmation method that uses over 86 specific genetic markers (Neogen, Lincoln, NE). Any samples that tested negative for either the *eae* gene or both *stx* genes in the RT-PCR virulence assay were assumed to be Top 7 STEC negative. All environmental samples that tested positive for *eae* and at least one *stx* gene (*stx_1_* or *stx_2_*) were submitted to Neogen for analysis. For animal samples (RAMS, hide, and udder), a maximum of three potential STEC samples from one source type (e.g., RAMS) in a single pen or cow herd were submitted to Neogen. If one on-plant sample was detected as potential STEC, all samples from that calf (RAMS, hide on farm, hide at processing plant, and preintervention carcass) were sent for NeoSEEK analysis regardless of screening results, in order to fully investigate potential transmission and contamination from calves transported from farms to processing plants. Selected DNA samples were shipped to Neogen dry ice.

### Data collection, database entry, and statistical analysis.

A Center 315 thermohygrometer (Center Technology, Taiwan) was used to record the humidity and temperature within and outside calf pens. Animal density was calculated by dividing the number of animals in each location (pen, calving paddock, and feedpad) by the area (square meters) of that location. Pen dimensions were measured manually, and calving paddock and feedpad areas were calculated using the area function on an eTrex GPS device (Garmin, Eastern Creek, Australia). All calves and cows sampled were given a hide cleanliness score (1 to 5) using the guidelines of the Food Standards Agency, where 1 indicates clean and dry and 5 indicates filthy and wet (24).

In order to evaluate potential transmission routes of STEC, positive Top 7 STEC (NeoSEEK) results from isolation sources (e.g., calving paddock or pen floor) were used as factors to evaluate associations with each outcome variable (listed in Table 8). If at least one sample was positive for a particular environmental source, the farm was considered positive for that source for the day.

**TABLE 8:** Outcome variables examined by statistical methods for “Top 7” STEC

Location	Variable	Description
On farm	Calf colonization (RAMS)	Recto-anal mucosal swab of calf on farm
	Calf hide on farm	Sponge swab of calf hide on farm
At processing plant	Calf hide at processing plant	Sponge swab of calf hide immediately post-stun on the processing line
	Preintervention carcass	Sponge swab of carcass after removal of the hide and before any decontamination intervention

Statistical analyses were performed in R (25) using a combination of univariate and multivariate regression models. The significance threshold was *P* < 0.05. Prevalence estimates with 95% confidence intervals were calculated from regression parameters using the *effects* package (http://socserv.socsci.mcmaster.ca/jfox/); in this method, a generalized linear model for each outcome variable was created using farm, period, and/or sample source as a random effect in order to account for our clustered study design.

A random forest model, constructed using the R package *randomForest* (26), was used to identify important factors associated with positive Top 7 STEC prevalence outcome variables.

A logistic mixed-effects model was used to evaluate the top 10 factors identified by the random forest output for each outcome variable, using “farm” as a random variable (effect). Factors were sequentially removed and checked for confounding versus other significant variables. Once a final model was determined, several biologically plausible risk factors that may have not been detected in the random forest output were tested in the model to reassess their importance.

### Bacterial isolation.

Isolation of STEC was attempted for all NeoSEEK-positive enrichment broth samples. Semithawed frozen glycerol enrichment broth samples (100 μl) were reenriched in mTSB medium for 18 h at 42°C, and then immunomagnetic separation (IMS) beads (Abraxis, Warminister, PA) were used following the manufacturer’s instructions with plating on three agars, as follows: CT-SMAC (for O157; Fort Richard Laboratories, Auckland, New Zealand), CT-RMAC (for O26; Fort Richard Laboratories, Auckland, New Zealand), and Rainbow agar O157 (for O45, O111, O103, O145; Biolog, Hayward, CA). CHROMagar STEC medium was also used for several serogroups (O45, O111, O103, and O145; CHROMagar Microbiology, Paris, France). In addition to reenrichment of enrichment broth samples, 20 μl of frozen glycerol enrichment broth was also plated directly onto the same four agars, without the IMS step.

### Whole-genome sequencing.

One isolate was randomly selected for whole-genome sequencing from each enrichment that yielded a Top 7 serogroup isolate (*n* = 40). DNA libraries were prepared at ^m^EpiLab using the Nextera XT protocol and submitted to New Zealand Genomics Limited (Massey Genome Service, Massey University, Palmerston North, New Zealand) for Illumina HiSeq sequencing. Raw sequence data were quality checked, assembled, and annotated using the Nullarbor bioinformatics pipeline (27). Virulence genes, antibiotic resistance genes, sequence type, and serotype were detected using the Center for Genomic Epidemiology pipeline (28). A core genome alignment was created, indicating variability (single-nucleotide polymorphisms [SNPs]) among genes shared by all genomes, with E. coli O26:H11 strain 11368 used as the reference for all serogroup O26 isolates and E. coli O157:H7 Sakai strain as the reference for all other serogroups. An accessory genome alignment based on presence and absence of accessory genes that were not present in all genomes was created with Roary version 3.11.3 (29). Distance (core genome SNP distance) and dissimilarity (accessory genome, virulence genes) matrices were created and evaluated using PERMANOVA and CLUSTER (PRIMER-E; Quest Research Limited, Auckland, New Zealand) with farm and isolation source as factors. Phylogenetic trees were visualized and annotated using interactive Tree Of Life (iTOL) software (30), and all figures were edited with Inkscape version 0.91 (https://inkscape.org/).

The NCBI Prokaryotic Genome Annotation Pipeline (PGAP) (https://github.com/ncbi/pgap) and SKESA version 2.2 were used to produce draft WGS assemblies, which are publicly available under these BioProject accession numbers PRJNA414662 and PRJNA396667.

### Data availability.

The data sets can be made available to researchers by contacting the corresponding author. All whole-genome sequence data (raw reads and assembled genomes) are publicly available in the NCBI database under BioProject accession numbers PRJNA414662 and PRJNA396667.

## Supplementary Material

Download

Download
