# Supplementary material for: Transmission Dynamics of Shiga Toxin-Producing Escherichia coli in New Zealand Cattle from Farm to Slaughter
Source: Appl Environ Microbiol. 2021 May 11;87(11):e02907-20. doi: 10.1128/AEM.02907-20 (PMC8208155; doi:10.1128/AEM.02907-20)
Supplement: Download [file AEM.02907-20_aem.02907-20.s0001.pdf]

Supplementary Material

Evaluating transmission dynamics of Shiga toxin-producing *E. coli* (STEC) in New Zealand cattle from farm to slaughter using advanced molecular diagnostics and genome sequencing

A. Springer Browne[1]*, Anne C. Midwinter[1], Helen Withers[2], Adrian L. Cookson[1,3], Patrick J. Biggs[1,4,5], Jonathan C. Marshall[1,5], Jackie Benschop[1], Steve Hathaway[2], Lynn Rogers[1], Shahista Nisa[1], Carter R. Hranac[1], Taylor Winkleman[1], Nigel P. French[1,4]

***Correspondence:** A. Springer Browne: drspringerbrowne@gmail.com

1 Supplementary Tables

Table S1: Generalized linear mixed model of effects of outcome variables on other outcome variables at the animal, shed, and farm level

Location	Hypothesis	Level	Parameter	Odds Ratio (95% CI)	p value
Farm	Calf colonization (RAMS) (outcome) is associated with calf hide contamination on farm	Animal: Positive prevalence in same animal	‘Top 7’ STEC	NC	0.08
		Shed: Presence or absence of a single positive in shed	‘Top 7’ STEC	12.1 (5.2, 28.3)	<0.00001
		Shed: Proportion of positives in shed	‘Top 7’ STEC	12.2 (5.2, 28.3)*	<0.0001
		Farm: Presence or absence of a single positive on that farm visit	‘Top 7’ STEC	14.4 (5.3, 29.1)	<0.00001
	Calf hide on farm contamination (outcome) is associated with calf colonization (RAMS)	Animal: Positive prevalence in same animal	‘Top 7’ STEC	1.40 (1.38, 1.41)	<0.000001
		Shed: Presence or absence of a single positive in shed	‘Top 7’ STEC	37.3 (4.7, 298)	<0.00001

		Shed: Proportion of positives in shed	‘Top 7’ STEC	2.01 (1.4, 3.0)*	<0.00001
		Farm: Presence or absence of a single positive on that farm visit	‘Top 7’ STEC	21.7 (2.6, 178)	0.0004
Processing plant	Calf hide contamination at processing plant (outcome) is associated with pre-intervention carcass contamination	Animal: Positive prevalence in same animal	‘Top 7’ STEC	NC	0.74
		Transport truck: Proportion of hide contamination of calves	‘Top 7’ STEC	NC	0.97
		Processing plant: Presence or absence of a single positive on that processing plant visit	‘Top 7’ STEC	NC	0.43

* per 10% increase in shed or truck prevalence

NC: not calculated due to non-significant finding

Table S2: Detection of 'Top 7' by NeoSEEK and success of retrieval of bacterial isolates

Serogroup	Samples detected as 'Top 7' STEC by NeoSEEK	Isolate recovered from sample*	STEC isolate recovered*	Overall STEC recovery
**O157**	13	4/13 (30.8%)	4/4 (100%)	4/13 (30.8%)
**O26**	56	25/56 (44.6%)	24/25 (96%)	24/56 (42.9%)
**O45**	15	1/15 (6.7%)	0/1 (0%)	0%
**O103**	70	4/70 (5.7%)	0/4 (0%)	0%
**O111**	5	0	0	0%
**O145**	45	6/45 (13.3%)	0/6 (0%)	0%

*At least one isolate was recovered from the frozen enrichment broth

Table S3: Prevalence of 'Top 7' STEC serogroup by NeoSEEK detection by farm (n=2580)

Farm ID	O103	O111	O121	O145	O157	O26	O45
**F1 (n=368)**	7% (n=25)	0	0	3% (n=10)	1% (n=4)	1% (n=5)	0.2% (n=1)
**F2 (n=483)**	1% (n=7)	0	0	2% (n=8)	0.2% (n=1)	0.6% (n=3)	0.2% (n=1)
**F3 (n=433)**	2% (n=9)	0	0	1% (n=6)	0.5% (n=2)	5% (n=20)	0.7% (n=3)
**F4 (n=317)**	0.6% (n=2)	2% (n=5)	0	0	0	0.3% (n=1)	0
**F5 (n=491)**	3% (n=15)	0	0	3% (n=14)	0	2% (n=9)	1% (n=7)
**F6 (n=488)**	3% (n=12)	0	0	1% (n=7)	1% (n=6)	4% (n=18)	0.6% (n=3)
**Total (n=2580)**	3% (n=70)	0.2% (n=5)	0	2% (n=45)	0.5% (n=13)	2% (n=56)	0.6% (n=15)

Supplementary Figures

Figure S1: RAxML phylogenetic tree of non-O26 serogroup E. coli core (a) and accessory (b) genomes annotated by farm, source, antibiotic resistance gene class, and virulence genes (n=105,136 SNPs detected; n=13,335 accessory genes detected)

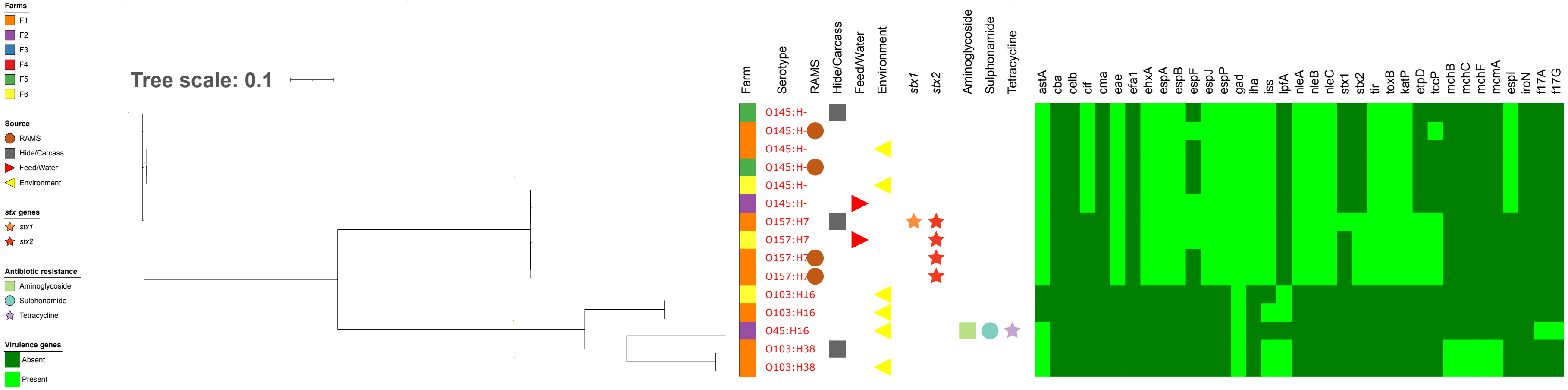

a: core genome

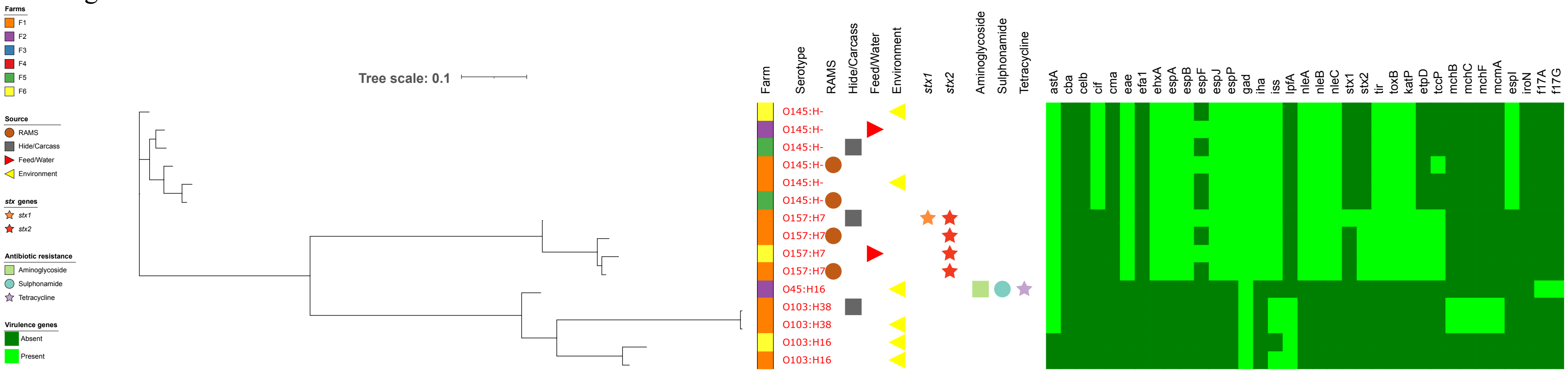

b: accessory genome
